# Using Search Engines to Find Online Medical Information

**DOI:** 10.1371/journal.pmed.0020228

**Published:** 2005-08-02

**Authors:** Mohammad Al-Ubaydli

## Abstract

Al-Ubaydli shares some useful tips for making the most of search engines.

Brewster Kahle, creator of the Internet Archive (www.archive.org)—a digital library of Internet sites and other cultural artifacts in digital form—has been inspirational in discussing the Internet's potential to become a modern Library of Alexandria. He campaigns for a resource that makes all of humanity's knowledge available to all of humanity.

The Internet certainly provides a number of resources for finding medical evidence. The Cochrane Collaboration (www.cochrane.org), for example, posts freely available abstracts of systematic reviews of health interventions (access to the full text of the reviews requires a fee). PubMed (www.ncbi.nlm.nih.gov/entrez/query.fcgi), the United States National Library of Medicine's search service, provides access to abstracts of articles in MEDLINE, PreMEDLINE, and other related databases. PubMed's MyNCBI feature provides useful filters such as “free full-text,” which shows papers for which the full text is available through the Internet, free of charge. The “HINARI” filter (www.nlm.nih.gov/pubs/techbull/jf05/jf05_myncbi.html#filters) shows papers for which the text is freely available to residents of a small number of developing world countries—those with a Gross National Product per capita below $1,000—who are part of the HINARI agreement (www.healthinternetwork.org). PubMed Central (www.pubmedcentral.nih.gov) is the US National Institutes of Health's free digital archive of the full text of biomedical and life sciences journal articles.

Yet, as many a doctor will point out, the bigger problem with medical knowledge today is not its paucity, but the difficulty of navigating what there is. Finding the right answer quickly for a patient is difficult, and perhaps nothing will replace a good medical librarian in finding that information.

The rise of the search engine Google (www.google.com), along with other freely available search engines, has made it easier to find information, although the clinical uses of Google have not been as well documented as those of PubMed [1]. Google will not point to the answer to every question, and often the articles it finds in response to your question are not freely available. But for many clinical scenarios, Google and other search engines can provide, quickly enough, an answer that is good enough. This article aims to provide tips that will help with these clinical scenarios, saving time that can be used with a medical librarian to answer more difficult problems.

## Search Engine Basics

Google provides a Web search engine—a tool that constantly indexes the expanding World Wide Web and allows you to search the index. Google's Web site is deceptively simple, designed to give you results quickly (Figure 1). Start by typing something into the text field and pressing the “Google Search” button. What you type in is the query, and what Google responds with is the results page.

**Figure 1:**
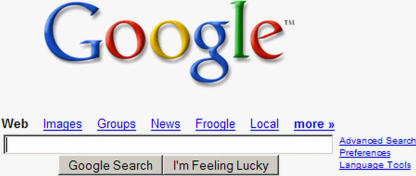
Google's Home Page

For example to learn about heart attacks, type “heart attack” as a query. Google's first page of results includes ten Web pages that cover heart attacks. The top right corner of Figure 2 shows that at the time of writing Google had found a total of about 20 million Web pages relevant to this query. Google ranks each of these Web pages by how many other Web pages provide links to them. This is the equivalent of the number of times a paper is cited; the more links a Web page gets, the greater the importance Google assigns to it, in the same way that the more citations authors receive, the greater the importance that academic institutions assign to their work.

**Figure 2:**
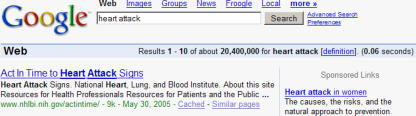
Results of the Search Term “Heart Attack”

Simply typing in the name of the medical condition is a good starting point, but it is a crude approach. For example, if your aim is to find information about thrombolysis for patients who have had a heart attack, then at least one of the 14.5 million pages that Google indexes in response to the query “heart attack” will be relevant. However, the first 20 pages Google produces say nothing about thrombolysis, and most of them are devoted to providing information for patients rather than clinicians. Rather than going through each of the millions of pages on heart attacks, it is faster to enter a slightly different query.

To find Web pages that are appropriate for clinicians, the query should include words that clinicians use. “Myocardial infarction” provides around 2.1 million results from Google, and some of the sites listed on the first page are likely to be relevant to clinicians (Figure 3). Being more specific with your search gives more specific results; the query “myocardial infarction thrombolysis” provides just 108,000 results, the first of which shows the guidelines on this topic [2] from the influential and well-respected National Institute for Clinical Excellence.

**Figure 3:**
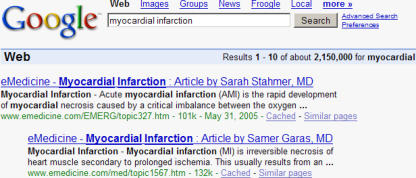
Results of the Search Term “Myocardial Infarction”

## Restricting the Web Sites Included in Your Search

Google has hidden depths. For example, adding “site:” to the end of a query restricts the search to certain Web sites. To focus on guidelines from Web sites maintained by the US federal government, type “myocardial infarction site:gov.” Using “site:nih.gov” focuses on the National Institutes of Health; “site:edu” restricts the search to American universities; “site:harvard.edu” to Harvard University; and “site:org” to nonprofit organizations.

Using “site:fr” as a search term will restrict your search to French Web sites, although not all French Web site URLs end with “fr” (for example the French Web site of Médecins Sans Frontières is www.paris.msf.org). There are similar search terms that you can use to restrict your search to particular countries, national health systems, or government agencies. For example, “site:nhs.uk” restricts the search to the British National Health Service, while “site:gv.kr” focuses on South Korean government Web sites.

Google also provides country-specific versions of its Web site. For example Google India (www.google.co.in) gives preferential ranking to Indian Web sites in its results and Google Kenya (www.google.co.ke) provides a Kiswahili interface. The full list of country-specific Google sites is available at www.google.com/language_tools.

## Other Google Features

At the top of the page (see Figure 1) are some of Google's other tools. For example, to find images of hip prostheses, type “hip prosthesis” as your search term and click the “Google Search” button. Clicking on the “Images” link will show a series of relevant photographs and diagrams that have been reduced in size (Figure 4). Clicking on any of these will display the image at full size. If the copyright owner of the image grants you permission, you can click on the image with the right-hand mouse button and choose to save it to your computer, then insert the image into your presentation or article.

**Figure 4:**
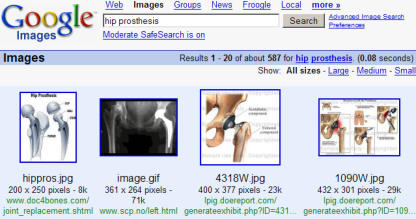
Results of a Google Images Search Using the Search Term “Hip Prosthesis”

The “News” link at the top of the page finds the latest news stories on a particular topic, and can be helpful for finding out what your patients have read in the lay press about a recent piece of medical research. The translation feature is useful for understanding content in languages that are not your own. On Google's English-language sites, the “Translate this page” link appears next to pages that are in languages other than English. Two books published by O'Reilly—*Google Hacks* [3] and the shorter *Google Pocket Guide* [4]—provide useful additional tips and guidance.

## Google Scholar

Perhaps the most clinically significant tool is Google Scholar (scholar.google.com), which is similar to PubMed in that it is a search engine that focuses on academic papers. In fact, many of the search results it returns are pages from the PubMed site. Google Scholar has a number of useful features that are not shared by PubMed. First, it is more comprehensive, indexing all academic fields, including non-biomedical ones. Second, and more importantly, the ranking mechanism is valuable. As with the rest of Google's technology, the pages are ranked based on the number of links that they receive. In the case of Google Scholar, “links” are citations from different papers. This means that review papers and seminal papers are most likely to top any list of results from a Google Scholar search.

Google Scholar is not a replacement for PubMed, since it lacks PubMed's precision searching. Furthermore, finding newer papers with Google Scholar is difficult; newer papers will not have been cited as much and so will be at the bottom of the results, and sorting by publication date is not possible.

## Other Search Engines

Google is the most popular search engine, but it is by no means the only one. Other search engines have different approaches with their own advantages. For example, Microsoft Network's query builder (search.msn.com) makes building complex queries easier. Yahoo's Creative Commons search feature (search.yahoo.com/cc) restricts searches to content (such as all of the content of the PLoS journals) that has been published under a Creative Commons license (www.creativecommons.org). These licenses are much less restrictive than the traditional “all rights reserved” copyright license. For example, if the content you have found (articles, photos, or images) is licensed under the Creative Commons Attribution License, you are legally entitled to reproduce it, distribute it, and make translations and derivative works, provided you cite the work properly.

The search engine Teoma (www.teoma.com) clusters search results according to different meanings of the words in the query. This clustering is useful because the medical meaning of some words, such as “hip,” is less commonly used than the non-medical meaning. Google lacks this clustering function. Finally, Vivisimo (www.vivisimo.com) can cluster results by subject (Figure 5). Its ClusterMed (www.clustermed.info) tool searches PubMed, while www.biometacluster.com simultaneously searches several relevant sources such as ChemBank and ClinicalTrials.gov. These are useful if you are searching for papers in a narrow specialty.

**Figure 5:**
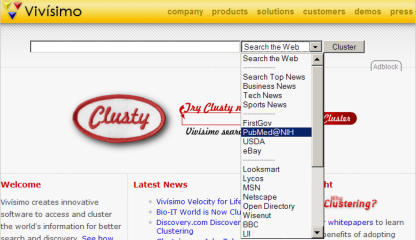
Vivisimo Searches the PubMed Database and Clusters the Results by Subject

## Conclusion

All of these freely available search engines have their limitations, and they rarely give you the perfect answer to your clinical query. But they do at least help to reduce the obstacles to finding medical information online. Kahle would certainly approve.
